# *CcNAC6* Acts as a Positive Regulator of Secondary Cell Wall Synthesis in Sudan Grass (*Sorghum sudanense* S.)

**DOI:** 10.3390/plants13101352

**Published:** 2024-05-14

**Authors:** Yanzhong Huang, Juanzi Wu, Jianyu Lin, Zhiwei Liu, Zhengfeng Mao, Chen Qian, Xiaoxian Zhong

**Affiliations:** 1 National Forage Breeding Innovation Base (JAAS), Institute of Animal Science, Jiangsu Academy of Agricultural Sciences, Key Laboratory for Saline-Alkali Soil Improvement and Utilization (Coastal Saline-Alkali Lands), Ministry of Agriculture and Rural Affairs, Nanjing 210014, China; huangyanzhong@163.com (Y.H.); jzwu2014@jaas.ac.cn (J.W.); 20120993@jaas.ac.cn (Z.L.); 2 National Center for Soybean Improvement, Key Laboratory of Biology and Genetics and Breeding for Soybean, Ministry of Agriculture, State Key Laboratory of Crop Genetics and Germplasm Enhancement, Nanjing Agricultural University, Nanjing 210095, China; jianyulin1996@126.com; 3 College of Agro-grassland Science, Nanjing Agricultural University, Nanjing 210095, China; 2020820042@stu.njau.edu.cn

**Keywords:** Sudan grass (*Sorghum sudanense* S.), secondary cell wall, bioinformatics analysis, *CcNAC6*, lignin

## Abstract

The degree of forage lignification is a key factor affecting its digestibility by ruminants such as cattle and sheep. Sudan grass (*Sorghum sudanense* S.) is a high-quality sorghum forage, and its lignocellulose is mostly stored in the secondary cell wall. However, the secondary cell wall synthesis mechanism of Sudan grass has not yet been studied in depth. To further study the secondary cell wall synthesis mechanism of Sudan grass using established transcriptome data, this study found that *CcNAC6*, a homologous gene of *Arabidopsis AtSND2*, is related to the secondary cell wall synthesis of Sudan grass. Accordingly, we constructed a *CcNAC6*-overexpressing line of *Arabidopsis* to investigate the function of the *CcNAC6* gene in secondary cell wall synthesis. The results showed that the overexpression of the *CcNAC6* gene could significantly increase the lignin content of *Arabidopsis*. Based on subcellular localization analysis, CcNAC6 is found in the nucleus. In addition, yeast two-hybridization screening showed that CcCP1, associated with secondary cell wall synthesis, can interact with CcNAC6. Therefore, the above results indicate that *CcNAC6* has a positive regulatory effect on the secondary cell wall synthesis of Sudan grass, and it is speculated that CcNAC6 may be the main regulator of the secondary cell wall synthesis of Sudan grass through its interaction with another regulatory protein, CcCP1. This study provides a theoretical basis and new genetic resources for the creation of new Sudan grass germplasm with a low lignin content.

## 1. Introduction

Sudan grass (*Sorghum sudanense* (Piper) Stapf.) is native to Africa and belongs to the genus Sorghum from the Gramineae family. It has numerous advantages, such as strong regeneration, potential for high grass yields, high nutritional value, and environmental stress tolerance. In livestock production, Sudan grass is not only suitable for fresh grass feeding but also for silage [1]. It has become an important feed crop in animal husbandry.

Structural carbohydrates such as cellulose, hemicellulose, and lignin in the cell walls of forage are the main nutritional components of Sudan grass. Ruminants such as cattle and sheep can digest and absorb cellulose and hemicellulose through their rumen microorganisms [2]. However, lignin in the cell wall is difficult to digest. Studies have shown that excessive lignin accumulation will not only affect livestock’s ability to digest forage grass but also plant growth [3]. Therefore, it is of great significance to increase forage yields and the digestibility of forage by ruminants such as cattle and sheep by studying the mechanism regulating the construction of the underlying secondary cell wall in forage grass. Studies have shown that plant NAC and MYB transcription factors are two-level regulatory factors that regulate secondary cell wall synthesis [4].

*NACs* (*NAM, ATAF1/2*, and *CUC2*) comprise one of the largest transcription factor gene families unique to plants. With more than 100 members, the constituents of this family are widely distributed in plants [5]. NAC proteins are composed of highly conserved N-terminals and variable C-terminals (TR). The N-terminal can be divided into five subdomains, and these different subdomains play different roles [6]. The N-terminal subdomain of the NAC protein domain can form a dimer, which binds to DNA to form a stable structure and regulates the expression of the target gene promoter. The C-terminal of the NAC protein domain has a highly variable transcriptional regulatory region and has different transcriptional characteristics under different conditions, thus activating or inhibiting different transcriptional processes [7]. In sorghum, the overexpression of the *GsNAC2* gene can promote plant growth by regulating glutathione metabolism [8]. In cabbage, *BcNAC056* regulates leaf senescence by activating the downstream expression of *BcSAG12* through interaction with *BcWRKY1* [9]. In peaches, *PpNAC1* and *PpNAC5* regulate fruit ripening and flavor by activating the expression of genes involved in sugar accumulation and organic acid degradation [10]. Additionally, plants can also enhance their resistance to abiotic stresses such as drought, freezing, and heavy metal ions through NAC transcription factors [11]. Therefore, NAC transcription factors play a crucial role in regulating plant growth and development, organ senescence, fruit ripening, abiotic stress responses, and various physiological functions.

*NAC* family transcription factors are the main switches regulating secondary cell wall thickening in plants [12]. A variety of NAC transcription factors are closely related to the construction of the secondary cell wall, including the *NST*, *SND*, and *VND NAC* transcription factors [13]. The redundancy of *NST1* and *NST2* regulates the deposition of the secondary cell wall in the inner wall cells of the anthers, and the overexpression of these two genes enhances secondary cell wall deposition and promotes anther dehiscence [14]. However, the *nst1/nst2* double mutation results in a loss of secondary cell wall formation in the anther walls of *Arabidopsis* [15]. 

*AtSND1* is specifically expressed in *Arabidopsis* wood fibers, and the inhibition of *AtSND1* expression can significantly inhibit secondary cell wall thickening in fiber cells [16]. The expression of *AtSND1* and *AtNST1* is also related to *AtATX1* (*H3K4-histonemethyltransferase*), which can regulate the expression of *AtSND1* and *AtNST1* based on H3K4me3 content [17]. Moreover, *AtSND2/3/4/5* may be redundantly regulated in secondary cell wall synthesis [18]. *AtSND1* can directly activate the expression of *AtMYB46* and *AtSND3* [19,20]. *AtSND2* activates the lignin synthesis-related genes *AtLAC4* and *AtLAC1*, a process that plays a role in lignification [21]. The overexpression of *AtSND2* can increase the thickness of the secondary cell wall, and the knockout of *AtSND2* can decrease the contents of the secondary cell wall [22]. In rice, SND2 has a negative effect on plants, with the overexpression of *OsSND2* leading to leaf curl [12]. 

The main accumulation sites for the secondary cell wall are the primary xylem duct and the metaxylem, which are formed by the regulation of AtVND6 and *AtVND7* [23]. In plants, *AtVND6* and *AtVND7* not only regulate the formation of the secondary cell wall but also programmed cell death (PCD) [24]. In Eucalyptus, the homologous gene *AtVND6* can be involved in the specific loss and PCD of the plant duct cell wall [25]. In addition, *AtVND7* is involved in pectin polysaccharide modification, which is key for distinguishing *VND* members from other NAC proteins [26]. In cotton, DELLA proteins can inhibit the positive regulatory effect of NAC transcription factors on secondary cell wall formation by directly interacting with *GhVND1* and *GhSND2* [27]. 

Moreover, these three types of NAC transcription factors can regulate the synthesis of the secondary cell wall by directly regulating downstream *MYB* transcription factors. However, not only do *MYBs* act as secondary transcription factors regulated by the *NAC* family, but they can also regulate the expression of *NAC* transcription factors in a feedback manner [28]. Studies have shown that *Arabidopsis AtMYB32* can inhibit the transcription expression of *AtSND1*, thus forming a negative-feedback regulatory pathway [29]. Existing studies have shown in model plants that *NST*, *SND*, and *VND NACs* act as positive factors in regulating secondary cell wall synthesis. However, studies on secondary cell wall lignin content in forage such as Sudan grass are relatively limited. Previously, in our laboratory, we transcriptome-sequenced the callus induced by the secondary cell wall of Sudan grass and screened the *CcNAC1* transcription factor, which was found to positively regulate the synthesis of plants’ secondary cell walls through functional verification [30]. However, other studies on lignin content in the secondary cell wall of Sudan grass have not been reported. We analyzed the differentially expressed *CcNAC* genes in the transcriptome data of the callus induced by the secondary cell wall of Sudan grass to further explore the key genes for lignin synthesis in the secondary cell wall of Sudan grass.

In this study, nine members of the *CcNAC* gene family, all with different expression levels, were identified based on data regarding the secondary cell wall-induced transcriptome of Sudan grass, and the identified *CcNAC* genes were analyzed via a bioinformatics analysis. This study provides a theoretical basis for further research on the function and molecular evolution of *CcNAC* genes related to the secondary cell wall of Sudan grass. In addition, this study also found and verified a gene homologous to *AtSND2*, *CcNAC6*, which was expressed in a pattern consistent with the trend of secondary cell wall content. These results not only reveal the regulatory mechanism of CcNAC6 in the secondary cell wall synthesis of Sudan grass but also provide a theoretical basis for further research on the role of *CcNAC* family members in secondary cell wall synthesis and gene resources for breeding new varieties of forage with low lignin content and high digestibility.

## 2. Results

### 2.1. Phylogenetic Analysis and Classification of CcNAC Genes

**Figure 1:**
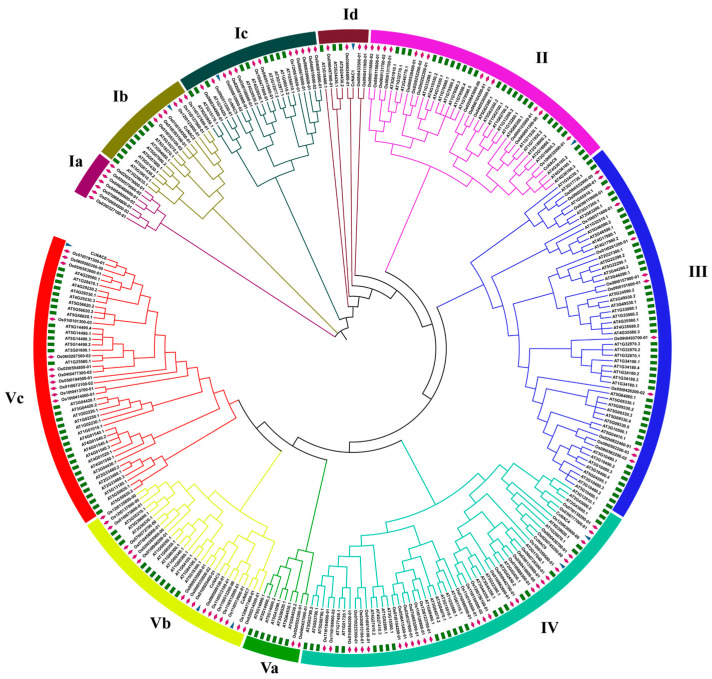
NAC proteins’ unrooted phylogenetic tree. The relationship between NAC proteins of Sudan grass, rice, and *Arabidopsis thaliana* was analyzed. NAC amino acid sequences were used to construct the neighbor-joining tree with parameters that included a Blosum62 cost matrix, the Jukes–Cantor model, global alignment, and a bootstrap value of 1000.

In order to study the genetic and biological relationship between Sudan grass and the NAC family members of *Arabidopsis thaliana* and rice, we selected 117 *Arabidopsis AtNAC* genes and 151 rice *OsNAC* genes as references. The phylogenetic trees of nine *CcNAC* genes whose expression was induced during the secondary cell wall synthesis of Sudan grass, and 117 *Arabidopsis AtNAC* genes, and 151 rice *OsNAC* genes were constructed using MEGA 11.0 software, and the evolutionary relationships among them were analyzed. According to the classification of the *AtNAC* gene family, the phylogenetic trees were divided into five groups: group I, group II, group III, group IV, and group V. Group I was further divided into I-a, I-b, I-c, and I-d, and group V was further divided into V-a, V-b, and V-c. As shown in Figure 1, the nine *CcNAC* genes were unevenly distributed in four groups, as there were three genes (*CcNAC1*, *CcNAC2*, and *CcNAC5*) in group I, one gene (*CcNAC8*) in group II, two genes (*CcNAC4* and *CcNAC9*) in group IV, three genes (*CcNAC3*, *CcNAC6*, and *CcNAC7*) in group V, and no *CcNAC* genes in group III. *CcNAC6* is highly homologous to the *Arabidopsis AtSND2* gene (*At4G28500*), and *CcNAC8* is highly homologous to the *Arabidopsis AtVND2* gene (*At4G361600*).

### 2.2. Multiple Sequence Alignment of CcNAC Genes

**Figure 2:**
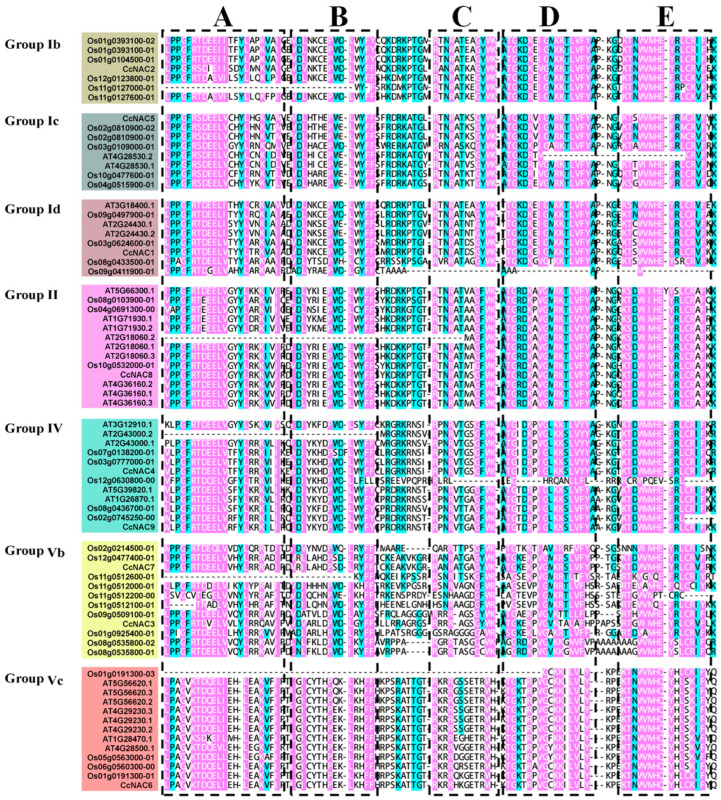
Sequence alignment of NAC proteins. Comparison of amino acid sequences of Sudan grass, rice, and *Arabidopsis thaliana*. A, NAC subdomain A; B, NAC subdomain B; C, NAC subdomain C; D, NAC subdomain D; E, NAC subdomain E.

NAC proteins have a conserved NAM domain for DNA binding, which is a key region for the biological functions of NAC proteins. Therefore, we focused on the differences between the domain sequences of the CcNAC members. The amino acid sequences of the NAC structural domains of the nine CcNACs were analyzed using multiple sequence alignment, and the characteristics of the NAC structural domains of the individual CcNACs were determined. We divided the NAC domains of the CcNACs into five subdomains, A–E (Figure 2). All nine CcNAC proteins contain five conserved subdomains within their amino terminus, indicating their strong sequence conservation during the evolutionary process.

### 2.3. CcNAC Promoter Motifs and Gene Structure Analysis

**Figure 3:**
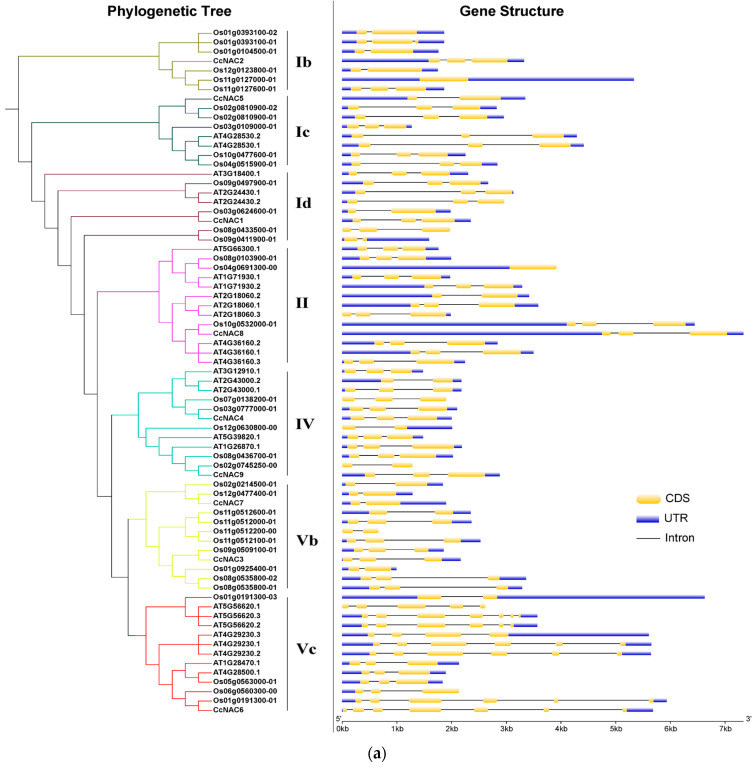

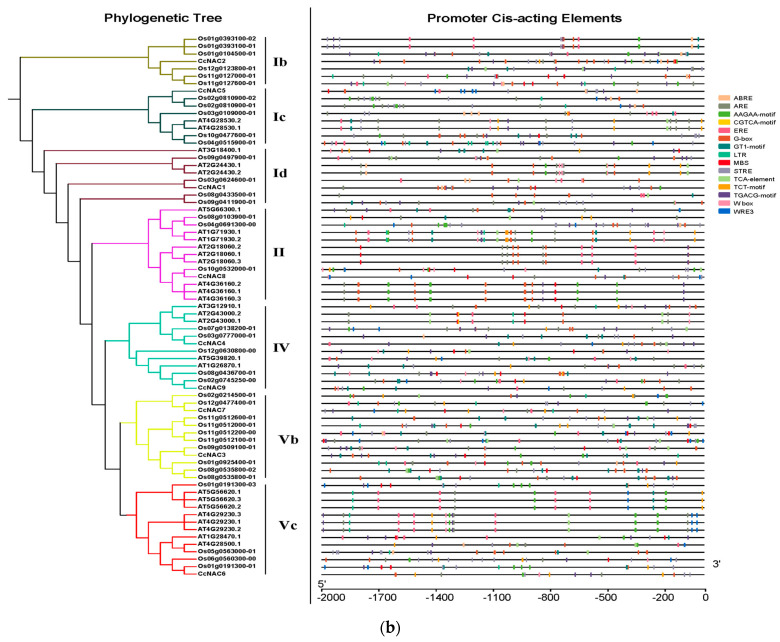
Gene structure analysis of NAC. (**a**) Analysis of full-length structure of Sudan grass, rice, and *Arabidopsis thaliana* NAC genes. (**b**) Analysis of upstream −2000 bp promoter cis-element of Sudan grass, rice, and *Arabidopsis thaliana* NAC genes.

To further explore the structure of the nine *CcNAC* genes, we analyzed their intron/exon compositions (Figure 3a). The number of introns ranged from one to six, and the number of exons ranged from two to seven, with *CcNAC6* containing the largest number of introns (six) and exons (seven). Most *CcNAC* genes contain three exons. Genes from the same subfamilies are similar in exon length, intron position, and number, indicating that the structure of NAC gene family members is relatively conserved.

In order to further understand the expression pattern of *CcNAC* genes, we analyzed the upstream 2000 bp promoter sequences of the nine *CcNAC* genes. As shown in Figure 3b, all upstream *CcNACs* have STER and TGACG motifs, and most upstream *CcNACs* mainly have cis-acting elements such as the G box, ARE, ABER, TCT motif, etc. The G box and TCT motif are related to light response, ABRE is related to abscisic acid (ABA) response, and the TGACG motif is related to jasmonic acid (JA) response. Based on transcriptome data, this is consistent with the expression of *CcNAC* genes in the light-induced callus of Sudan grass.

### 2.4. Gene Duplication and Collinearity Analysis of CcNACs

**Figure 4:**
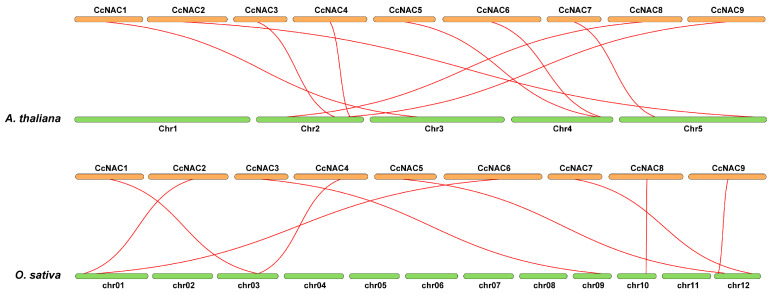
Interspecies collinearity analysis of Sudan grass *CcNAC* genes.

To further explore the evolutionary interrelationships of the NAC genes in different species, we performed interspecies collinearity analysis among *CcNACs* of *Arabidopsis thaliana* and rice (Figure 4). The nine *CcNAC* family members of Sudan grass have homologous relationships with *Arabidopsis* and rice, but the homologous genes are unevenly distributed on chromosomes. In *Arabidopsis*, there are four genes distributed on chromosome 2, two genes each on chromosomes 4 and 5, one gene on chromosome 3, and no homologous genes distributed on chromosome 1. In rice, there are three genes distributed on chromosome 12, two genes on chromosomes 1 and 3, one gene on chromosomes 9 and 10, and no homologous genes on chromosomes 2, 4, 5, 6, 7, 8, and 11. By comparison, it was found that the *CcNAC* gene related to secondary cell wall synthesis in Sudan grass has a homologous gene in both *Arabidopsis* and rice, so these nine genes were not found to be genes that specifically regulate secondary cell wall synthesis in Sudan grass.

### 2.5. Overexpression of CcNAC6 Increases Lignin Content in A. thaliana Plants

**Figure 5:**
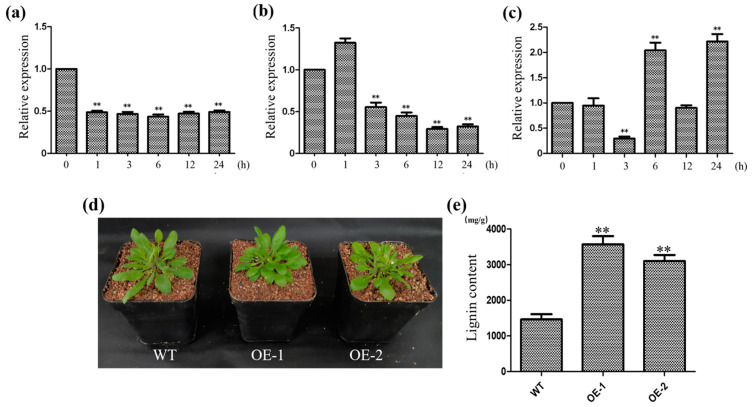
*CcNAC6* positively regulates secondary cell wall synthesis. (**a**) The expression specificity of the *CcNAC6* gene was analyzed by qRT-PCR after ABA treatment. (**b**) The expression specificity of the *CcNAC6* gene was analyzed by qRT-PCR after JA treatment. (**c**) The expression specificity of the *CcNAC6* gene was analyzed by qRT-PCR after BR treatment. (**d**) Performance of wild-type (WT) plants and *CcNAC6* overexpression lines (OE1 and OE2) under normal conditions. (**e**) Lignin content of wild-type plants and *CcNAC6* overexpression lines after 2 months of planting *(n* = 3); over 30 plants in each line were used for survival rate analysis. The data represent the means ± SEs. **, *p* < 0.01 (Student’s *t* test).

We selected *CcNAC6*, a homologous gene of *AtSND2*, to study the function of *CcNAC* genes in regulating the lignin content of Sudan grass. Our analysis of promoter elements showed that *CcNAC* promoters contain ABA and JA elements, and a previous transcriptome analysis found that *CcNAC* genes are also induced by brassinosteroids (BRs). So, we treated Sudan grass with ABA, JA, and BR hormones and found that the *CcNAC6* gene was significantly down-regulated within one day after the ABA and JA treatments (Figure 5a,b), while it was significantly up-regulated within one day after the BR treatment (Figure 5c). 

We created *Arabidopsis* lines (OE1 and OE2) with overexpression of *CcNAC6* to study its role in the synthesis of secondary cell walls. The growth rate of the *CcNAC6*-overexpressing lines (OE1 and OE2) did not show a significant decrease compared to the wild type (WT) (Figure 5d). Lignin levels were measured in both the overexpressing lines and the WT, revealing that the lignin content in the overexpressing lines (OE1 and OE2) was significantly higher (2.1 times and 2.4 times, respectively) than that in the WT (Figure 5e).

### 2.6. CcNAC6 Encodes a Nuclear Localization Protein

**Figure 6:**
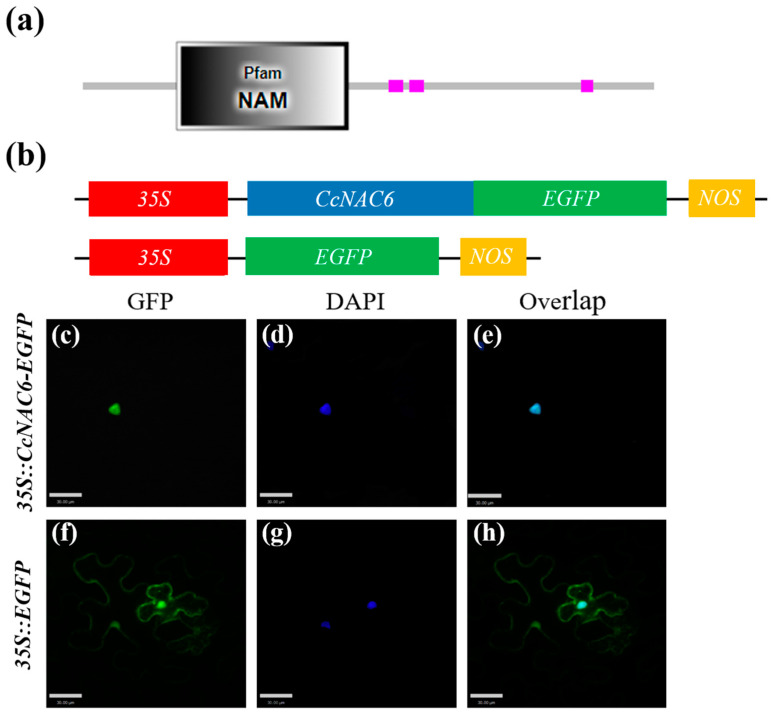
Subcellular localization of CcNAC6 in tobacco cell. (**a**) Amino acid sequence analysis of CcNAC6. (**b**) Construction of CcNAC6 subcellular localization vector. (**c**–**e**) *35S::CcNAC6-GFP* fluorescence images in *N. benthamiana* cell. (**f**–**h**) Fluorescence images of tobacco cells expressing *35S::GFP* were obtained. *N. benthamiana* leaves were subjected to transient infiltration with A. tumefaciens *GV3101* carrying a vector that expressed either *35S::GFP* or *35S::CcNAC6-GFP*. The confocal microscope from Zeiss was used to capture all the images after agroinfiltration for a duration of 48 h. DAPI images indicate nuclear staining. Scale bars are 50 μm.

In order to investigate the mechanism by which the CcNAC6 protein regulates the synthesis of plant secondary cell walls, we conducted a study on the functional regions of CcNAC6 to determine its subcellular localization. Our prediction based on the NAC protein domain analysis indicated that CcNAC6 consists of 469 amino acids, with amino acids 77–218 belonging to the NAM domain (Figure 6a). It was predicted that CcNAC6 is localized in the nucleus.

To confirm this prediction, we constructed a fusion expression vector, *35S::CcNAC6-EGFP*, where *EGFP* is fused with *CcNAC6* and controlled by the *35S* promoter (Figure 6b). We performed transient expression experiments in *N. benthamiana* leaves using both fusion expression vectors and empty vectors to examine the subcellular localization of CcNAC6. DAPI staining was used as a nuclear marker. The green fluorescent protein (GFP) signal was visualized using laser confocal microscopy. Our results revealed that a GFP signal from an empty vector was observed in the cytoplasm, cell membrane, and nucleus (Figure 6f–h). In contrast, only the fusion protein expressed by *35S::CcNAC6-EGFP* was detected exclusively in the nucleus (Figure 6c–e). These findings provide evidence supporting the idea that CcNAC6 localizes within the nucleus.

### 2.7. CcNAC6 Physically Interacts with CcCP1

**Figure 7:**
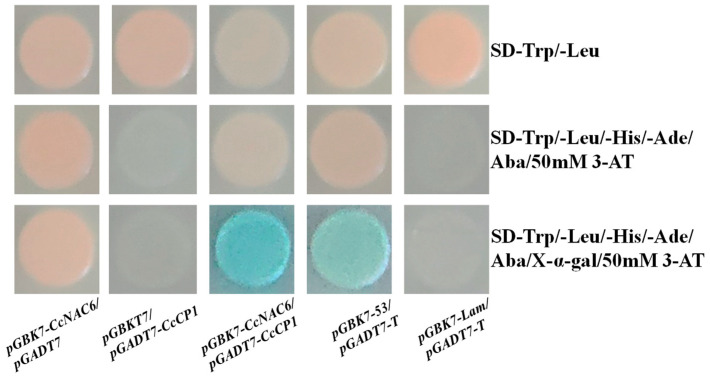
CcNAC6 physically interacts with CcCP1. Protein interaction between CcNAC6 and CcCP1 was confirmed through yeast two-hybrid analysis using a selective growth method combined with an X-α-gal activity assay. Negative controls included yeast cells transformed separately with constructs such as *pGBKT7-Lam*/*pGADT7-T*, *pGBKT7*/*pGADT7-CcCP1*, or *pGBKT7-CcNAC6*/*pGADT7*. A positive control consisted of yeast cells transformed with constructs named *pGBKT7-53*/*pGADT7-T*.

To further understand the molecular interactions and mechanism of CcNAC6 in the regulation of secondary cell wall synthesis in Sudan grass, the yeast library screening technique was utilized to determine CcNAC6’s interactions with other proteins present inside the cell. It was observed that CcNAC6 interacts with the cysteine proteinase CcCP1 (Figure 7). It has been reported that cysteine proteinase is involved in programmed cell death (PCD) in xylem cells and fibrocytes in plant vascular tissues during secondary wall thickening.

As demonstrated, the co-transfection of yeast cells with *pGBKT7-CcNAC6* and *pGADT7* empty vectors, *pGBKT7* empty vectors and *pGADT7-CcCP1*, or *pGBKT7-Lam* and *pGADT7-T* did not result in a blue coloration in the selection medium, indicating an absence of protein–protein interaction (PPI). However, when both *pGBKT7-CcNAC6* and *pGADT7-CcCP1* were co-transfected into yeast cells, a blue color appeared, suggesting that CcNAC6 interacts with CcCP1 within yeast cells. For this study’s positive controls, yeast cells were co-transfected with *pGBKT7-53* and *pGADT7-T*.

## 3. Discussion

The structural carbohydrates contained in the cell wall of Sudan grass, such as cellulose, hemicellulose, and lignin, comprise its main nutrients. Ruminants such as cattle and sheep digest and absorb cellulose and hemicellulose in lignocellulosic fibers through microorganisms in the rumen [31], but lignin in the cell wall cannot be digested and also affects the digestion of other nutrients [3]. Therefore, in order to improve the digestibility of forage by ruminants such as cattle and sheep, it is of great significance to explore the synthesis of the key genes of each component of the secondary cell wall in order to reduce the contents of indigestible substances. Moreover, improving forage digestibility will also increase livestock’s carrying capacity per unit area of land. Reducing the content of indigestible substances in forage through breeding and cultivation strategies would have a significant economic impact on agriculture.

A lack of reliable genomic information and genetic databases has limited the research on Sudan grass. At present, relevant gene information can only be obtained by transcriptome sequencing. Through transcriptome analysis, *CcNAC1* was previously identified as a positive regulator of secondary wall synthesis in Sudan grass [30], but a detailed analysis of all nine distinct *CcNAC* genes has not yet been reported. 

Bioinformatics has become an important means for the effective processing, mining, and analysis of biological information data [32]. In this study, through bioinformatics analysis, it was found that the *CcNAC6* gene is highly homologous to *Arabidopsis AtSND2*, and following gene function verification, we also found that the *CcNAC6* gene can significantly increase the lignin content when overexpressed in plants (Figure 5d,e).

Through phylogenetic tree analysis with *Arabidopsis* and rice, it was found that not all of the nine *CcNAC* genes were *NST*, *SND*, and *VND* genes reported to be associated with secondary cell wall synthesis. Only *CcNAC6* was highly homologous to *AtSND2* (*At4G28500*), and *CcNAC8* was highly homologous to *AtVND2* (Figure 1). However, the homologous genes of *Arabidopsis thaliana* among these nine genes are all related to cell wall synthesis [33,34,35]. This may be because the other seven genes do not directly regulate the synthesis of secondary cell walls in Sudan grass. It is possible that, in addition to *NAC* transcription factors that can directly regulate secondary cell wall synthesis, such as *NST*, *SND*, and *VND*, other types of *NACs* may indirectly regulate secondary cell wall synthesis in plants [36]. In *Arabidopsis thaliana*, *AtVNI2* can inhibit its transcriptional activity by interacting with *AtVND7*, thus inhibiting the differentiation of xylem conduit cells [37]. *AtVNI1* and *AtANAC103* can regulate the secondary cell wall synthesis of *Arabidopsis thaliana* by interacting with *AtVND7* [38].

Although the *NAC* gene family is a large gene family in plants, the structures of the genes in the *NAC* gene family are very different [39]. Our analysis of the domains of the nine *CcNAC* genes found that all of them could be divided into five subdomains (Figure 2). Although there were some differences in amino acids in the conserved domains of the NACs and homologous genes, the similarity between the nine NAC protein domains and those of the homologous genes may be due to the fact that NAC genes with similar functions also have similar protein domains [40]. The study of homologous genes in model plants also provides a reference for further research on the function of CcNACs in the secondary cell wall synthesis of Sudan grass. 

Most *CcNAC* genes are composed of three exons. The gene with the highest number of exons was found to be *CcNAC6*, which has seven exons (Figure 3a). Although the protein domains of the *CcNAC* genes are similar to those of *Arabidopsis*’s and rice’s homologous genes, the exon compositions of the gene introns are very different (Figure 3a). The structure of the *CcNAC* gene of Sudan grass is similar to that of rice. This may be because Sudan grass and rice are monocotyledonous plants.

Our analysis of the nine *CcNAC* genes and their homologous gene promoter elements in Sudan grass showed that there were significant differences in the number of homologous gene promoter elements in Sudan grass, *Arabidopsis*, and rice, as well as their promoter distributions, but the types of elements were similar (Figure 3b). This result means that these genes are similarly induced and involved in similar biological pathways in different species. However, due to the differences in the number and location of the elements, the expression level of these genes may be different, and while their functions in the biological pathway may be similar, the roles they play may be different [41]. 

In *CcNAC* promoters, the promoter elements are related to light, ABA, and JA. Our studies have shown that both light and BRs can induce the synthesis of the secondary cell wall of Sudan grass [30], but our analysis of the promoter elements found that no elements are related to BRs (Figure 3b). Previous studies have shown that BRs and ABA antagonistically regulate plant development, and in rice, the two hormones synergistically regulate rice leaf inclination through the ABI3-OsGSR1 pathway [42]. Moreover, in *Arabidopsis*, AtABI1 and AtABI2 can regulate the response to drought stress by regulating the activity of the BR signaling pathway negative regulator *AtBIN2* [43]. In addition, BRs are also involved in abiotic stress responses such as salt tolerance and cold resistance induced by ABA [44,45]. JA also antagonizes BRs to regulate plant development, and JA can inhibit BR biosynthesis. In rice, these two hormones can regulate the lamina joint inclination through the OsBRI1-OsGSK2 pathway [46]. Moreover, in *Arabidopsis*, JA and BRs can synergistically regulate anthocyanin accumulation and root elongation in *Arabidopsis* seedlings [47,48]. 

In Sudan grass, ABA and JA may inhibit *CcNAC* gene expression, while BRs can relieve the inhibition of *CcNAC* expression by these two hormones. In our study, the *CcNAC6* gene was significantly down-regulated within one day after the ABA and JA treatments (Figure 5a,b), while it was significantly up-regulated within one day after the BR treatment (Figure 5c), which also confirmed the previous speculation.

NAC transcription factors, as primary transcription factors, play a very important role in the regulation of secondary cell wall synthesis in plants. *CcNAC6* is a homologous gene for *AtSND2* in *Arabidopsis* [49]. The overexpression of *CcNAC6* in *Arabidopsis* can significantly increase the lignin content in *Arabidopsis* plants (Figure 5d,e). The function and energy ratio of the *AtSND2* gene are relatively preserved in grasses and woody plants, and the overexpression of *AtSND2* in Eucalyptus also increases the thickness of the secondary walls of Eucalyptus fibrocytes [50]. 

In this study, CcNAC6 was used as a bait protein to screen the secondary cell wall yeast library of Sudan grass. CcNAC6 can interact with CcCP1 (Figure 6). Cysteine proteinases are induced by the programmed cell death (PCD) of pipe cells [51]. PCD occurs in xylem cells in plant vascular tissue. Cysteine protease and other hydrolases degrade the contents of the pipe cells and form cavities while the secondary wall thickens [52]. Therefore, *CcNAC6* may be related to xylem pipe formation in Sudan grass. The released CcCP1 interacts with CcNAC6 so that, like *Arabidopsis AtSND2*, *CcNAC6* activates the expression of the *LAC4* and *LAC1* genes and positively regulates secondary cell wall synthesis in fibrocytes. In rice, a gene homologous to *AtSND2*, *OsSND2*, can positively regulate secondary wall formation. OsSND2 can also directly bind to several MYB gene promoters, such as *OsMYB61*, which regulates secondary wall biosynthesis [12]. Therefore, CcNAC6, which interacts with CcCP1, may also directly bind downstream *MYB* transcription factors to regulate the secondary cell wall synthesis of Sudan grass. *CcNAC6* is, therefore, a switching factor that regulates secondary wall biosynthesis in plants.

The content of secondary cell wall lignin in Sudan grass is very important for the digestibility of ruminants such as cattle and sheep, but the molecular mechanism of secondary cell wall lignin synthesis in herbage has been limited to model plants, leading to limited research on secondary cell wall lignin content in forage grass such as Sudan grass [30]. Due to the lack of a complete genome sequence of Sudan grass, it was difficult to find genes related to lignin synthesis through reverse genetics, and only relevant genes could be discovered by transcriptome sequencing. Moreover, the transgenic system of Sudan grass was not perfect, and the discovered genes could not be functionally verified in Sudan grass. In the future, after obtaining the complete genome sequence of Sudan grass, more key genes for secondary cell wall lignin synthesis of Sudan grass would be mined through homologous cloning and map cloning, and after the maturity of the Sudan grass transgenic system, the expression of key genes for secondary cell wall lignin synthesis of Sudan grass could be regulated by gene editing, and new varieties of Sudan grass with low lignin content and high digestibility could be cultivated.

## 4. Materials and Methods

### 4.1. Data Used in This Study

Based on the transcriptome sequencing data of Sudan grass (PRJNA1093012) [30], the transcription factor database plant TFdb (http://planttfdb.gao-lab.org/index.php (accessed on 5 April 2022)) was used to classify the transcription factor family. *NAC* transcription factor family genes were selected, BlastX comparison was performed again, and full-length *NAC* genes with complete open reading frame (ORF) were screened by ORF Finder analysis. Using ExPASy (https://prosite.expasy.org/prosite.html (accessed on 10 April 2022)), we analyzed the structure of the gene-encoding protein domains. We used the TAIR online website (https://www.arabidopsis.org (accessed on 12 April 2022)) to download the *AtNACs* in the *Arabidopsis thaliana* genome and protein sequences, and the Phytozome online website (Phytozome (doe. Gov)) to download the *OsNACs* in the rice genome and protein sequences. 

### 4.2. Construction of Phylogenetic Tree

The amino acid sequences of *Arabidopsis* and rice NAC proteins were compared with those of Sudan grass. ClustalW in MEGA 7.0 software was applied for multi-sequence comparison, and the neighbor-joining method (NJ) of MEGA 7 was applied to construct and analyze the NAC phylogenetic tree of Palmatum Rhubarb. The bootstraps value was 1000. We used the ChiPlot online software (https://www.chiplot.online (accessed on 1 May 2022)) to beautify the evolutionary tree [53].

### 4.3. Analysis of Protein Conserved Domain and Collinear Relationship

DNAMAN 9.0 software was used to compare the amino acid sequences of Sudan grass CcNACs and download the *Arabidopsis* and rice NAC proteins [54]. 

Using Ensembl Plants (http://plants.ensembl.org/index.html (accessed on 20 May 2022)), we downloaded *Arabidopsis*’s and rice’s chromosome length and the whole-genome sequence information (GFF3 and fasta format). The MCscanX tool of Tbtools was used to analyze the collinearity between Sudan grass and two other plants, and the NAC gene of Sudan grass was highlighted [55]. 

### 4.4. Analysis of Gene Structure and Promoter Cis-Acting Elements of Gene Family

According to the obtained NAC genome sequence, the gene structure of the *Arabidopsis*, rice, and Sudan grass *NAC* gene families was mapped using GSDS 2.0 (http://gsds.cbi.pku.edu.cn/ (accessed on 5 June 2022)), and the 2000 bp sequences upstream of the transcription initiation of *NAC* genes were selected and submitted to PlantCARE (https://bioinformatics.psb.ugent.be/webtools/plantcare/html (accessed on 15 June 2022)) to predict the composition of cis-acting elements. The results were sorted, and only the hormone elements and regulatory elements on the justice chain were retained. Finally, visualization operations were performed on the TBtools software (TBtools-Ⅱ(Toolbox for Biologists) v2.016).

### 4.5. Plant Materials and Growth Conditions

Sudan grass variety “Sumu No. 3” was used as the material for hormone treatment and sampling. Sudan grass seeds were sown in soil and cultivated in greenhouses at 26 °C for long days (16/8 h light/dark) with a relative humidity of 70%. After 2 weeks of growth, Sudan grass was treated with ABA, JA, and BR hormones. A total of 90 Sudan grass plants with the same growth rate were selected for each hormone treatment and divided into 3 replicates. Next, 5 Sudan grass plants were taken as samples at 0, 1, 3, 6, 12, and 24 h after treatment with 150 μM ABA [56], 100 μM JA [57], and 2 μM BRs [30], wrapped in aluminum foil, frozen in liquid nitrogen, and stored at −80 °C.

*Arabidopsis thaliana* variety “Columbia-0” was used as material to verify gene function. *Arabidopsis* seeds were cultured in soil, first vernalized at 4 °C for 5 days, and then grew in a controlled growth chamber at 22 °C for long days (16/8 h light/dark) with a relative humidity of 70%. Two months after planting, 90 *Arabidopsis* plants were divided into 3 replicates using overexpression and wild type for phenotype comparison, and 10 *Arabidopsis* plants were selected as samples in each replicate to determine the lignin content of *Arabidopsis*.

### 4.6. Lignin Content Measurement Method

The lignin levels in various strains of *Arabidopsis thaliana* were assessed using the lignin assay kit. Wild-type (WT) lines and *CcNAC6* overexpression lines (OE1 and OE2) were cultivated for 2 months under normal growth conditions. Subsequently, the lignin content was determined utilizing the lignin content determination kit (AKSU010U, Boxbio, Beijing, China). Three independent replicates were conducted, and the standard error (SE) was calculated.

### 4.7. Quantitative Real-Time PCR (qRT–PCR) Verification

Gene-specific primers for qRT-PCR were designed using GenScript Real-time PCR primer design tool (https://www.genscript.com/tools/real-time-pcr-taqman-primer-designtool (accessed on 15 June 2023)) and are listed in Supplemental Table S1. Template cDNAs were synthesized from 1.0 μg total RNA using the Prime Script™ RT Reagent Kit (TaKaRa, Kyoto, Japan). ChamQ Universal SYBR qPCR Master Mix (Q711, Vazyme, Nanjing, China) was used as the labeling agent, and *CcEIF4a* was used as the internal reference gene. These reactions were performed using an Applied Biosystems™ QuantStudio™ 5 Real-Time PCR apparatus. The sample period threshold (CT) of each template was standardized according to the internal reference gene control primer response, and the relative change in gene expression was analyzed by the 2^−ΔΔCT^ method [5]. To ensure statistical confidence, three independent biological replicates were performed for each sample.

### 4.8. Subcellular Localization of CcNAC6 Protein

To identify the subcellular location of the CcNAC6 protein, we used the SMART online software (http://smart.embl-heidelberg.de/smart/set_mode.cgi?NORMAL=1 (accessed on 5 September 2023)) to predict the CcNAC6 protein domain by analyzing its amino acid sequence. To verify the localization of the CcNAC6 protein in plant cells, the CDS of the *CcNAC6* gene without the terminator was cloned into the *pBinGFP4* vector to construct a fusion vector following the manufacturer’s protocol (Figure 6b). Then, we injected the constructed and empty vectors into tobacco leaves. Leaf fluorescence was observed under a Zeiss LSM 880 Upright Confocal Microscope (Carl Zeiss, Oberkochen, Baden-Württemberg, Germany) at 48−72 h post-inoculation. The excitation wavelengths were 488 nm and 405 nm for green fluorescent protein (GFP) and 4′,6-diamidino-2-phenylindole (DAPI), respectively.

### 4.9. CcNAC6 Overexpression in Sudan Grass Plants

The *pTF101.1* vector was utilized to incorporate the complete coding sequence (CDS) of the *CcNAC6* gene, which was placed under the regulation of the *CaMV 35S* promoter (*35S::CcNAC6*). Following confirmation of the vector sequence through sequencing, *Arabidopsis thaliana* cultivar Columbia-0 plants were transformed with the recombinant *pTF101.1-CcNAC6* plasmid vector using *Agrobacterium tumefaciens* strain EHA101. The presence of transgenic plants expressing the desired traits was confirmed by PCR amplification of both a selected marker gene (*bar*) and the *35S* promoter. Phenotypic evaluation of homozygous transgenic lines was carried out in the T_3_ generation.

### 4.10. Yeast Two-Hybrid Assay

By utilizing a Y2H system (Takara, Kyoto, Japan), we conducted a screening of a Sudan grass callus yeast library provided by Yuanbao Biotech (Nanjing, China). This screening yielded numerous proteins that exhibited interactions with the CcNAC6 protein. Given that there is no existing report on NAC’s interaction with cysteine proteinase (CP1), our focus shifted towards investigating its interaction with CcCP1 instead. To facilitate this study, we cloned the full coding sequences of both *CcNAC6* and *CcCP1* into vectors, specifically *pGBKT7* and *pGADT7*, respectively, resulting in recombinant plasmids named *pGBKT7-CcNAC6* and *pGADT7-CcCP1*. These constructs were then co-transformed into Y2H Gold yeast strain cells before being streaked onto various media, including those containing SD/−Trp/−Leu plates, SD/−Trp/−Leu/−Ade/−His + Aba + 50 mM 3-AT plates, or even SD/−Trp/−Leu/−Ade/−His + Aba + 50 mM 3-AT+X-α-Gal plates.

The primers used in this study were from Genscript (Nanjing, China), and gene sequencing in this study was performed by General-Biol (Chuzhou, China)

## 5. Conclusions

In this study, according to the transcriptomes induced by the secondary cell wall of Sudan grass in the previous period, the nine differentially expressed *CcNAC* genes were analyzed through bioinformatics, and it was found that the *CcNAC6* gene was highly homologous to *AtSND2*, a key gene for lignin synthesis in *Arabidopsis thaliana*. Therefore, this gene was selected for further study. It was found that the overexpression of this gene could significantly increase lignin content in plants. In addition, using the yeast two-hybridization (Y2H) method, we found that CcNAC6 and cysteine proteinase 1 (CcCP1) interact, and cysteine proteinase 1 was found to be involved in programmed cell death (PCD), so *CcNAC6* may regulate secondary cell wall thickening by regulating PCD. Therefore, our results suggest that *CcNAC6* is a key regulator of secondary cell wall synthesis in Sudan grass.

## Data Availability

Data are contained within the article and Supplementary Materials.

## Supplementary Materials

The following supporting information can be downloaded at https://www.mdpi.com/article/10.3390/plants13101352/s1: Table S1. Primer pairs in this study.
