# Supplementary material for: CcNAC6 Acts as a Positive Regulator of Secondary Cell Wall Synthesis in Sudan Grass (Sorghum sudanense S.)
Source: Plants (Basel). 2024 May 14;13(10):1352. doi: 10.3390/plants13101352 (PMC11125125; doi:10.3390/plants13101352)
Supplement: Supplementary file 1 [file plants-13-01352-s001.zip › plants-2968258-supplementary.pdf]

**Supplemental Table S1**

**Primers used for clone gene**

| Gene | Sequence of primer pairs (5'-3') |
|---|---|
| *CcNAC6* | TCTGTCCAGCTCCAGCATCA/AGTGGTTGTGGTTAGTGTTCCTT |

**Primers used for quantitative real-time PCR**

| Gene | Sequence of primer pairs (5'-3') |
|---|---|
| *CcNAC6* | CAGGTGTGACGAGGGATGGC/CTCGTCTCGCCCTTGTGGAC |
| *CcEIF4a* | AGGATTGGCACCAGAAGGGT/CACATCAAGCCCCTTGCAGA |

**Primers used to construct vector for plant expression and transgenic plants detection**

| Gene | Sequence of primer pairs (5'-3') |
|---|---|
| *CcNAC6* | GTGTTAACGGAATTCGAGCTCATGCCCATCGCAGCAAGA/ |
| | GGATCCCCGGGTACCGAGCTCCTATCCATGATGATCTTGGTTGTCA |

**Primers used to construct vector for subcellular localization**

| Gene | Sequence of primer pairs (5'-3') |
|---|---|
| *CcNAC6* | ATTTACGAACGATAGGGTACCATGCCCATCGCAGCAAGA/ |
| | TCCGTCGACCCCGGGGGTACCTCCATGATGATCTTGGTTGTCAGG |

**Primers used to construct vector for yeast two-hybrid**

| Gene | Sequence of primer pairs (5'-3') |
|---|---|
| *CcNAC6* | TGGCCATGGAGGCCGAATTCATGCCCATCGCAGCAAGA / |
| | CGCTGCAGGTCGACGGATCCCTATCCATGATGATCTTGGTTGTCA |
| *CcCP1* | GCCATGGAGGCCAGTGAATTCATGGCTCATCGCGTTCTCC/ |
| | ATGCCCACCCGGGTGGAATTCCTACTCCTTCGAGGTGCGGA |
